# Color Stability, Surface Gloss, Surface Roughness, and Wettability of Material Jetting 3D-Printed Denture Material Under Various Surface Treatments

**DOI:** 10.3390/dj13050220

**Published:** 2025-05-20

**Authors:** Toshiki Nagai, Amal Alfaraj, Wei-Shao Lin

**Affiliations:** 1 Department of Prosthodontics, Indiana University School of Dentistry, Indianapolis, IN 46202, USA; 2 Department of Prosthodontics and Dental Implantology, College of Dentistry, King Faisal University, Al Ahsa 31982, Saudi Arabia; asalfara@iu.edu

**Keywords:** MJT, Polyjet, home care, esthetics

## Abstract

**Objectives:** To examine the effects of surface treatments on the color stability, surface roughness, surface gloss, and wettability of monolithic polychromatic material jetting (MJT) 3D-printed denture material. **Material and Methods:** Twenty-one color variants of the same denture material (TrueDent; Stratasys, Eden, MN, USA) underwent two surface treatments (polishing only or polishing and glazing), creating 42 study groups with a total of 420 samples (n = 10 per group). The samples were manufactured using a PolyJet 3D printer (J5 DentaJet; Stratasys, Eden, MN, USA), a type of MJT 3D printer. Color measurements were taken with a digital spectrophotometer before and after the surface treatments, and quantitative color differences (ΔE_00_ and ΔC*) were calculated using the CIE2000 system. Comparisons of ΔE_00_ were made against the 50%:50% acceptability threshold (AT) of 1.8 and the 50%:50% perceptibility threshold (PT) of 0.8 for tooth shade, as well as the 50%:50% PT of 1.72 and the 50%:50% AT of 4.08 for gingival (pink) shade. After surface treatment, the gloss was measured using a glossmeter, surface roughness was measured with optical profilometry, and wettability was measured by contact angle measurements using an optical tensiometer. The significance of surface treatment on color changes for each color variant was evaluated using one-sided, one-sample *t*-tests against the AT and PT. The effects of surface treatment on surface gloss, surface roughness, contact angle, and ΔC* were analyzed using *t*-tests for each color variant. Pairwise comparisons between groups were made using Fisher’s Protected Least Significant Differences (α = 0.05). **Results:** In most cases, glazing caused the color change (ΔE_00_) to exceed the AT and PT, with a few exceptions. Most materials exhibited a more vibrant (more saturated) appearance and statistically higher chroma, with glazed surface treatments compared to polished ones, though there were some exceptions. For all materials, the glazed samples had significantly higher gloss units than the polished ones (*p* < 0.0001). Additionally, all materials showed significantly higher surface roughness in glazed samples compared to polished ones (*p* < 0.0001 for most). The polished samples had significantly higher contact angles (*p* < 0.0001 for most). **Conclusions:** Surface treatments significantly influenced the color, surface gloss, surface roughness, and wettability of MJT 3D-printed denture materials. Glazing led to increased chroma and gloss and produced more hydrophilic surfaces, although it also increased surface roughness. These results highlight the importance of surface treatment selection in optimizing the clinical performance of MJT-fabricated dentures.

## 1. Introduction

Since the pioneering work by Maeda in 1994, digital technologies have significantly advanced the fabrication of complete dentures, particularly through the application of additive manufacturing (3D printing) [1]. While subtractive manufacturing methods such as milling have become widely adopted due to their superior dimensional stability, enhanced retention, and precise tissue adaptation [2,3,4,5], they present notable drawbacks—including considerable material waste, high fabrication costs, limited ability to mill complex geometries [6], and restricted digital tooth libraries.

Additive manufacturing technologies, especially Vat Photopolymerization (VPP) technologies like stereolithography (SLA), digital light processing (DLP), and liquid crystal display (LCD), have become common in prosthodontics [7]. A growing number of studies have extensively evaluated the properties of 3D-printed resins produced by these technologies, focusing not only on mechanical strength (e.g., flexural strength), but also surface characteristics, solubility, translucency, sorption, and color stability [8,9,10]. However, there is still a significant lack of data on denture materials printed using material jetting (MJT) technology.

**Figure 1:**
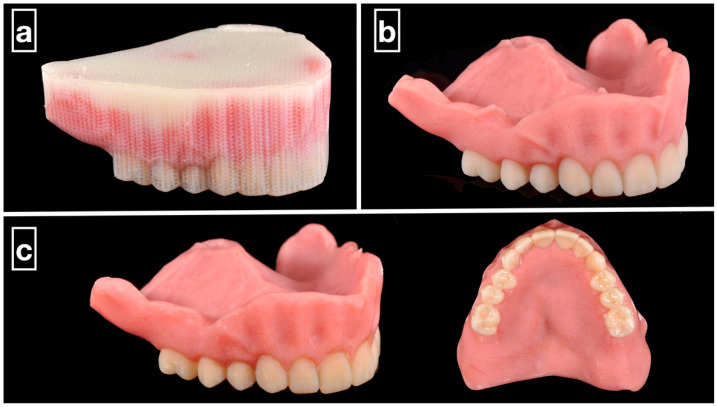
Clinical example of 3D-printed monolithic polychromatic complete dentures. (**a**) The MJT 3D-printed denture before support structure removal. (**b**) Support structure removed from the prosthesis using waterjet. (**c**) The prosthesis polished and glazed.

MJT is a distinctive additive manufacturing technique in which photopolymer droplets are selectively deposited and cured layer-by-layer using ultraviolet (UV) light [11]. Commercial systems such as PolyJet (Stratasys, Eden Prairie, MN, USA) and MultiJet (3DSystems; Rock Hill, SC, USA) [12] enable the fabrication of monolithic polychromatic complete dentures with high dimensional accuracy and smooth surface finishes (Figure 1) [13,14]. This capability eliminates the need for the additional bonding or extrinsic characterization often required in SLA- or DLP-based systems [6].

The standardized testing of denture base materials is essential for the comparative assessment of key properties that influence denture functionality and durability. Surface roughness, defined as small indentations or irregularities on the material surface, impacts wettability, adhesion quality, and gloss. Ideally, surface roughness should remain below 0.2 μm for denture base materials to minimize microbial adhesion [15], plaque accumulation, halitosis, staining, and patient discomfort [16,17,18]. Surface coatings have been shown to reduce the increase in roughness following mechanical wear, such as brushing, when compared to uncoated surfaces [19]. Optiglaze (GC America Inc., Alsip, IL, USA), a light-polymerizing glaze containing titanium dioxide nanoparticles, has been widely used in dentistry to enhance the surface properties of polymeric materials [20,21]. Surface gloss, which contributes to the esthetic acceptance of dentures, tends to decline with material deterioration and color changes [22,23]. Previous studies have reported that milled denture bases exhibit lower surface roughness, while the 3D-printed bases often demonstrate higher gloss values than conventionally polished auto-polymerizing resin bases [24]. Color remains a challenging factor in the additive manufacturing of dentures, and while the optical properties of dental prostheses have been extensively studied [25], there is lack of research on the color and gloss characteristics of MJT-printed denture materials—particularly under different surface treatments.

Therefore, the objective of this study was to investigate the effects of two surface treatment protocols—polishing only and polishing followed by glazing—on the color stability, surface gloss, surface roughness, and wettability of MJT 3D-printed denture materials. The null hypothesis was that surface treatments would not significantly affect any of these surface characteristics.

## 2. Materials and Methods

**Figure 2:**
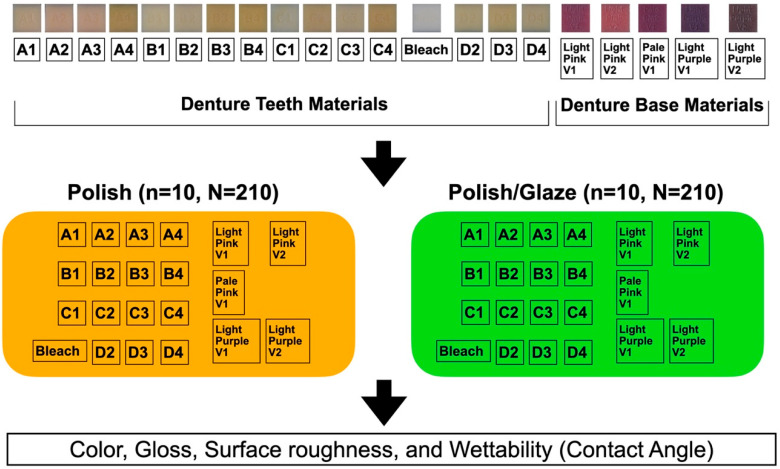
Study design.

**Figure 3:**
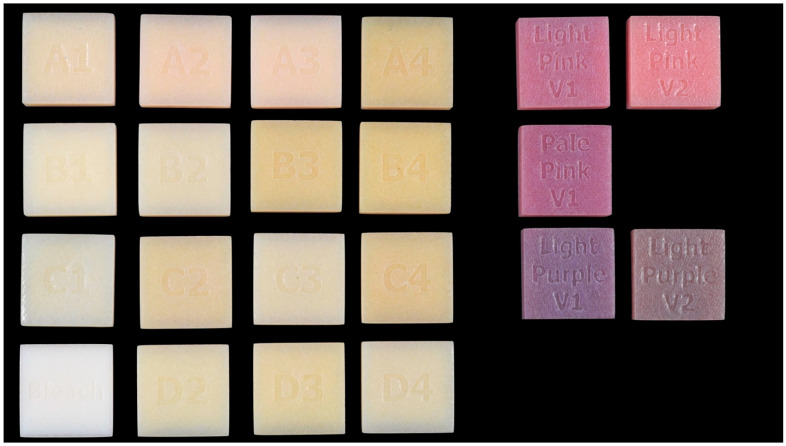
Study samples as manufactured.

**Figure 4:**
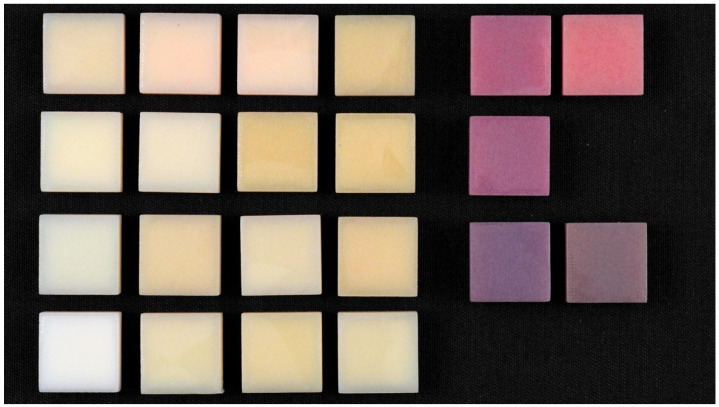
Polished samples.

**Figure 5:**
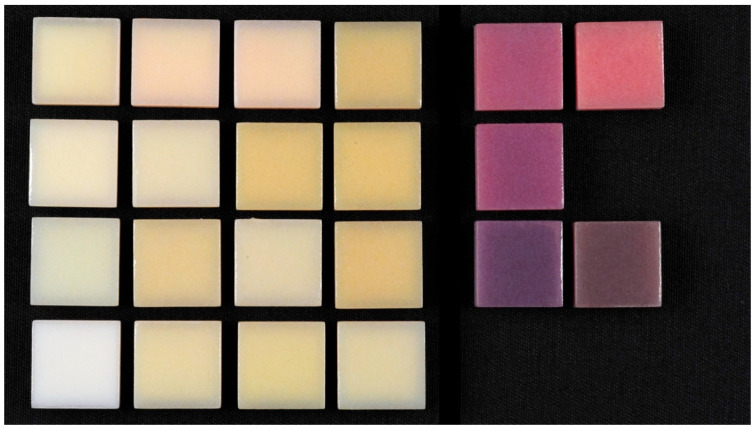
Glazed samples.

The study design is shown in Figure 2. The MJT 3D-printed denture materials of 21 different colors (TrueDent; Stratasys, Eden, MN, USA) and 2 surface treatments were combined as 42 study groups (Figure 3). A total of 420 samples, with dimensions of 12.0 mm × 12.0 mm × 6.0 mm, were used (n = 10 per study group). The sample dimensions were decided based on if each sample could physically fit into all the testing devices required for this study. All study samples were fabricated by certified manufacturers (Stratasys, Eden, MN, USA) using a PolyJet 3D printer (J5 DentaJet; Stratasys, Eden, MN, USA). All samples received polishing as the first step of surface treatment. One surface of the specimens was polished using a polishing machine (Stuers Rotopol 31-Rotoforce 3; Spectrographic Limited, Leeds, UK) under water irrigation and 300 rotations per minute. Grinding paper (Silicon Carbide grinding Paper; Stuers LLC, Cleveland, OH, USA) with 2000 grit was used to polish the samples. One cycle of 30 s was used to polish each sample (Figure 4). For glazing, all samples were polished utilizing the same protocol as the polished samples followed by adding one layer of a nano-filled, light-polymerizing protective glaze coating (Optiglaze; GC America Inc., Alsip, IL, USA) on the polished surface, and were polymerized for 3 min at 30 °C using a light-polymerization unit (Otoflash G171; NK Optik GmbH, Baierbrunn, Germany). Disposable microbrushes (Benda Brush Regular; Centrix Inc., Shelton, CT, USA), composed of non-lining, non-absorbent fibers, were used to apply a thin glaze coating (Figure 5). One investigator (NT) ensured full coverage on each sample.

Color measurements were performed by one investigator (TN) using a contact-type digital spectrophotometer (CM-2600d Spectrophotometer; Konica Minolta Healthcare Americas Inc., Wayne, NJ, USA) to calculate the color precision of the study samples. The small aperture SAV (∅3 mm) was used, and the spectrophotometer was calibrated using both zero calibration (measuring with nothing in front of the spectrophotometer measurement port for at least 3 ft) and white calibration (White Calibration Plate CM-A145; Konica Minolta Healthcare Americas Inc.). The color measurements were obtained under the following conditions: UV (100%), illumination (D65), mask/gloss (S/SCI + SCE), and observer angle (10%). Three color measurements were taken for each study sample, and the mean of these 3 measurements was recorded and used for further statistical analyses. A custom-made silicon mold (Exalence putty; GC American, Alsip, IL, USA) was utilized to position the study samples during color measurements to reduce the possibility of external light reflection, ensure contact, and maintain the light angle during the study. The CIEDE2000 formula was used to determine the color difference ΔE_00_ between polished and glazed samples. The hue, chroma, and lightness corrections were included in the calculations as follows:

$$\;\Delta E_{00}=\sqrt{{\left(\frac{∆L’}{k_{L}S_{L}}\right)}^{2}+{\left(\frac{∆C’}{k_{C}S_{C}}\right)}^{2}+{\left(\frac{∆H’}{k_{H}S_{H}}\right)}^{2}+{\;R}_{T}\left(\frac{∆C’}{k_{C}S_{C}}\right)\left(\frac{∆H’}{k_{H}S_{H}}\right)}$$

The 50:50% perceptibility threshold (PT) and 50:50% acceptability threshold (AT) values for the ΔE_00_ color difference in this study were defined as 0.8 and 1.8, respectively, for tooth shade [26,27], as well as PT = 1.72 and AT = 4.08 for gingival (pink) shade [28]. The ΔChroma (ΔC*) values were also recorded during the ΔE_00_ calculations for a subsequent comparison.

A glossmeter (Novo-Curve Glossmeter; Rhopoint Americans Inc., Troy, MI, USA) was used to determine the surface gloss in gloss units (GU). Before testing, the glossmeter was calibrated with a standard tile (assigned a gloss unit value of 93.8 gloss units under the measurement angle of 60°). The center of the specimen was aligned with the center of the glossmeter at the first measurement. Then, for the second measurement, the sample was rotated 90° clockwise. The exact same process was repeated for the third and fourth gloss measurements. The mean of the four measurements was calculated and recorded as the representative surface gloss unit for the sample.

Surface roughness was assessed using an optical profilometer (Proscan 2000; Scantron, Taunton, UK) (n = 420) under a spectral type of sensor (S5/03; Scantron Industrial Products Ltd., Taunton, UK) with a 5 mm stand-off (distance from the target in mm that the sensor needed to be at to take measurements), 300 μm measuring range, and 0.010 μm resolution. The step size was set at 0.01 mm, and the number of steps was set at 100 on the *X*-axis and 100 on the *Y*-axis. The scans were analyzed using software (Proscan Application software v. 2.0.17; Scantron Industrial Products Ltd.) with an auto-leveling function applied. The surface roughness was measured at the center of each specimen and expressed in μm.

Wettability was assessed by measuring contact angles via the sessile drop method using an optical tensiometer (Theta Lite; Biolin Scientific, Gothenburg, Sweden) within a controlled environment (room temperature maintained at 23 ± 2 °C and humidity at 45%). In brief, 5 µL of millipore water was applied to the sample surface. Real-time images were captured for 5 s to conduct live analysis. Mean contact angles were derived from two measurements along the imaginary line connecting the reference notches of each sample. Contact angles can define surface wettability as hydrophilic (0° ≤ θ ≤ 45°) or hydrophobic (90° ≤ θ ≤ 180°) [29]. To visualize the surface of the samples (A1P, A1G, Light Pink V2P, and Light Pink V2G) under FESEM, the samples were immersed in hexamethyldisilazane (Electron Microscopy Sciences, Hatfield, PA, USA) and gold coated with 2000× magnification.

With a sample size of 10 specimens per group, the study had 80% power to detect the effect sizes between any two groups of 1.32 for any outcome. The significance of the color changes on a 50:50% acceptability threshold (AT) of 1.8 and a 50:50% perceptibility threshold (PT) of 0.8 for tooth shade, as well as an AT of 1.72 and PT of 4.08 for gingival shade, were evaluated using one-sided one-sample *t*-tests. The effects of polished and glazed surface treatments on surface gloss, surface roughness, contact angles, and ΔC* were analyzed using a *t*-test for each material. The pairwise comparisons between groups were made using Fisher’s Protected Least Significant Differences to adjust for the familywise errors. A two-sided 5% significance level was used for all tests in the statistic software program (SAS version 9.4; SAS Institute Inc., Cary, NC, USA) (α = 0.05).

## 3. Results

**Figure 6:**
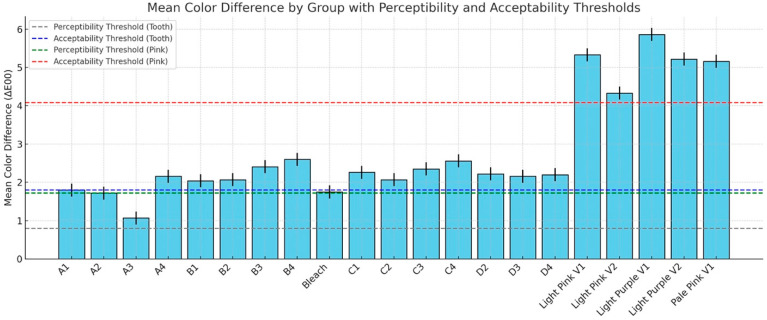
Color difference (ΔE_00_) between polished and glazed samples, compared against the 50:50% perceptibility threshold (PT = 0.8) and acceptability threshold (AT = 1.8) for tooth shade, as well as PT = 1.72 and AT = 4.08 for (gingival) pink shade.

**Figure 7:**
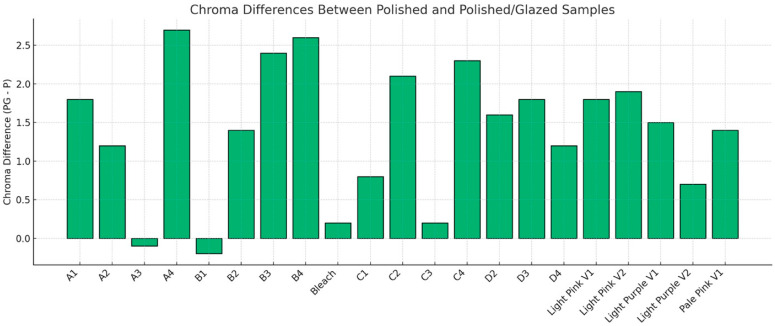
Chroma differences (ΔC*) between polished-only (P) and polished and glazed (PG) samples. Positive values (PG−P) indicate higher chroma in PG groups, while negative values indicate higher chroma in P groups. Increased chroma corresponds to a more vibrant and saturated appearance.

The descriptive statistics for the color difference (ΔE_00_) between polished and glazed samples are shown in Figure 6, and the results are compared to the 50:50% PT of 0.8 and 50:50% AT of 1.8 for tooth shade as well as PT = 1.72 and AT = 4.08 for gingival shade. In most cases, glazing caused the color to change beyond the AT and PT. For the PT, only the A3 material stayed within the PT. For the AT, only the A1, A2, A3, B1, B2, C2, and Bleach materials were within the AT. To further compare specific aspects of color changes with different surface treatments, ΔC* was evaluated. The results of ΔC* are presented in Figure 7. With polished and glazed surface treatments, most of the materials showed statistically higher chroma (more vibrant and saturated appearance) with glazed surface treatment than polished ones, though there were a few exceptions. The A3, B1, Bleach, C1, and C3 showed no significant chroma changes between the polished and glazed treatments.

**Figure 8:**
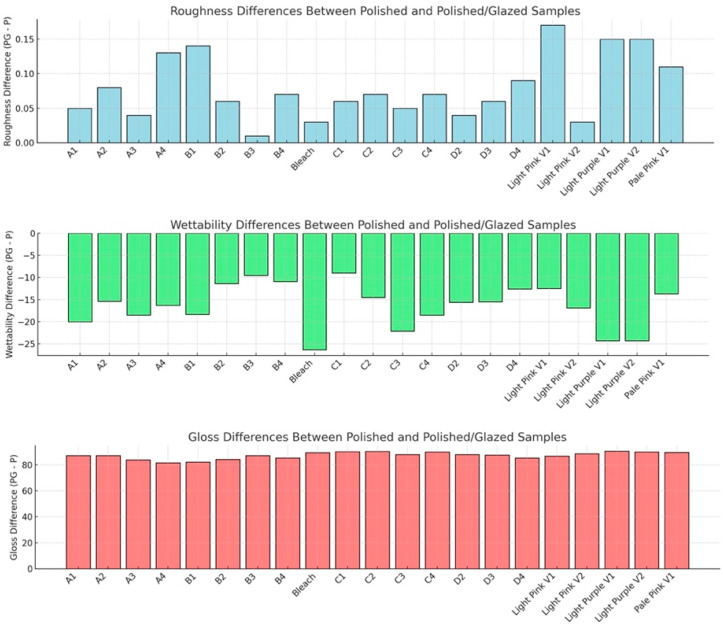
Results for surface roughness (mean ± standard deviation, μm), wettability (contact angle, mean ± standard deviation, degrees), and gloss (gloss units, mean ± standard deviation, GU). P represents polishing-only groups, and PG represents polishing and glazing groups. Roughness difference (PG−P): positive values indicate PG samples are rougher than P. Wettability difference (PG−P): negative values indicate that PG samples exhibit lower wettability (i.e., higher contact angle) than P. Gloss difference (PG−P): positive values indicate that PG samples have higher gloss than P.

**Figure 9:**
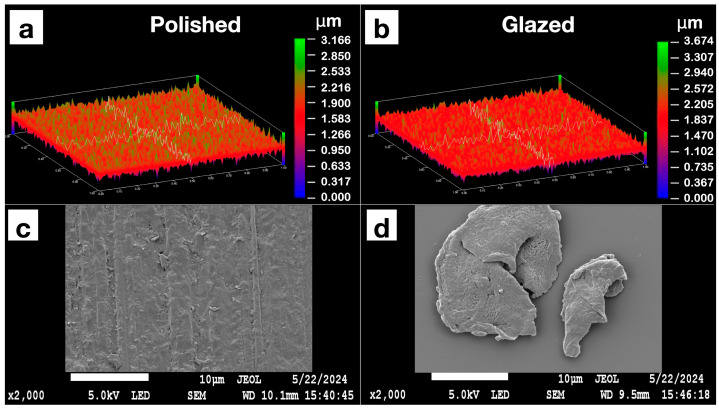
Representative images of the Light Pink V2 sample’s surface roughness with polished or glazed surface treatments under an optical profilometer are presented in (**a**,**b**). The representative scanning electron microscope (SEM) images are presented in (**c**,**d**). (**a**) Polished sample profilometer image; (**b**) glazed sample profilometer image; (**c**) polished sample SEM image; (**d**) glazed sample SEM image.

**Figure 10:**
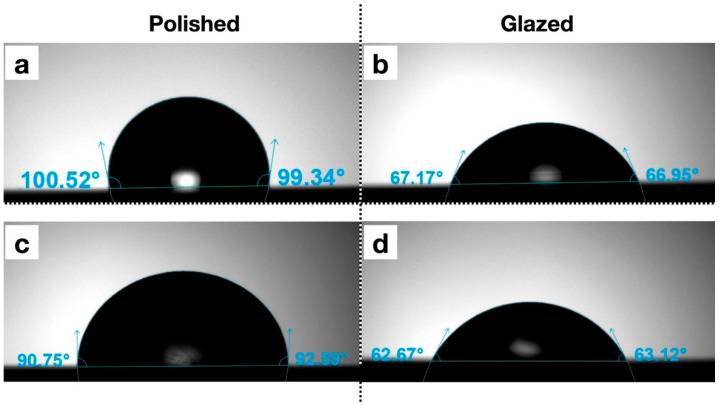
Representative images of contact angles with different surface treatments. (**a**) A1 polished; (**b**) A1 glazed; (**c**) Light Pink V2 polished; (**d**) Light Pink V2 glazed.

The statistical results and corresponding graphical representations of surface roughness, wettability, and gloss are presented in Figure 8. Except for B3 (*p* = 0.4514), all materials showed significantly higher surface roughness in glazed samples than the polished ones (*p* < 0.0001 for most). The representative scanned images of surface roughness with different surface treatments (polished vs. glazed) are presented in Figure 9a,b. The representative SEM images from polished and glazed samples are presented in Figure 9c,d. The representative images of contact angles with different surface treatments are presented in Figure 10. For all materials, the polished samples had significantly higher contact angles (*p* < 0.0001 for most) and were significantly more hydrophobic than glazed ones. For all materials, the glazed samples had significantly higher gloss units than the polished ones (*p* < 0.0001).

## 4. Discussion

The null hypotheses were rejected, showing that surface treatments affected the color, surface gloss, surface roughness, and wettability of MJT 3D-printed denture materials. In the present study, surface treatment significantly affected the ΔE_00_ between polished and glazed samples, and glazing caused the color change beyond the AT and PT (Figure 6). Although studies have shown that both polishing and glazing significantly enhance color stability [27,30,31], no research has yet evaluated the color change (ΔE_00_) caused by the surface treatment in MJT 3D-printed denture material. Previous research has primarily investigated the accuracy of surface adaptation [32].

While prior investigations have assessed surface roughness and color stability in SLA- or DLP-printed resins, particularly under simulated aging, such as brushing and thermocycling, these studies have not included MJT materials. For instance, Çakmak et al. evaluated the effects of simulated brushing and thermocycling on DLP-printed, milled, and heat-polymerized denture base resins. Their results revealed that brushing and thermal aging can lead to significant changes in surface roughness and perceptible color differences (ΔE₀₀ > 1.72), although all remained within acceptable limits (ΔE₀₀ < 4.08) [33]. These outcomes, however, are limited to other 3D-printing technologies and cannot be directly extrapolated to MJT-based materials, highlighting the novelty and importance of the current study. The glazing procedure involved applying a light-polymerized coating that reduced surface porosity by filling in micropores and micro defects [34]. A nano-filled protective coating (Optiglaze) was applied to minimize microleakage in this study, and the literature indicated that it significantly reduces color change [27,35,36]. However, only the A3 material remained within the PT, while for the AT, only the A1, A2, A3, B1, B2, C2, Bleach materials, and Light Pink V2 fell within the AT range in this study after surface glazing, with no statistically significant differences. This outcome may be attributed to material composition, properties, and potential manual procedure variation during the glazing procedure, although standardized manufacturing was employed.

To further compare the specific parameters of color changes with different surface treatments, ΔC* was evaluated in this study (Figure 7). Most materials exhibited a statistically higher chroma (ΔC*) with glazed surface treatment than polished treatment. Higher chroma resulted in a more vibrant and saturated appearance of the study samples. One possible explanation for this outcome could be the generally high sorption and solubility characteristics of 3D-printed denture material [37]. The current MJT 3D-printing denture material may absorb the glazing material, leading to a color shift toward higher chroma [38]. To the best of the authors’ knowledge, however, no studies are currently available that specifically address color, solubility, and sorption differences based on the surface treatment for MJT 3D-printing denture materials. This study suggests that a glazed MJT 3D-printed shade guide may be necessary during the shade selection procedure when fabricating MJT 3D-printed dentures with a planned glazed surface treatment to ensure optimal color-matching outcomes.

Due to various limitations, CIELab does not always provide accurate and consistent measurements of color differences. To address these issues, the CIE introduced CIEDE2000 in 2000. The improvements in CIEDE2000 include correcting for non-uniform color spacing, more accurately calculating hue and chroma differences, and accounting for the perceptual non-uniformity of the human visual system. As a result, CIEDE2000 offers a more precise and consistent measurement of color differences, particularly for colors that are not equally spaced and for larger color differences [38,39,40,41,42,43]. Therefore, the CIEDE2000 system was used in this study to calculate quantitative measurements of color differences (ΔE_00_). The ΔE_00_ values were compared with the PT and AT. The 50%:50% PT represents the color difference detectable by 50% of observers, while the 50%:50% AT indicates the color difference considered acceptable by 50% of observers [38]. In this study, the 50%:50% AT was set at ΔE_00_ = 0.8 and the 50%:50% PT at ΔE_00_ = 1.8 for tooth shade, as well as 50%:50% PT = 1.72 and 50%:50% AT = 4.08 for gingival (pink) shade [26,27,28,38,39,40,41,42,43].

The present study revealed that all materials showed significantly higher surface roughness in glazed samples than in polished ones, comparable to a previous study [44]. However, this result does not align with a few other studies, which either showed no effect of glazing on surface roughness [19] or indicated that glazing produced a smoother surface compared to non-glazed surfaces [44,45]. This inconsistency may be attributed to manual variations in glazing protocol and denture base material differences, as most studies have focused on polymethyl methacrylate (PMMA) [20] and hybrid resins [46,47]. The representative scanned images of surface roughness and the SEM images with different surface treatments (polished vs. glazed) are presented in Figure 10. These images suggest that glazing may create debris on the surface at the microscopic level, despite the general observation that glazing typically results in a smoother appearance to the unaided eye [45].

In this study, for all materials, the polished samples had significantly higher contact angles and were significantly more hydrophobic than glazed ones (Figure 10). Wettability (contact angle), along with surface charge, roughness, and topography, are essential factors affecting the colonization and biofilm formation by fungi and bacteria on denture base materials [48,49,50,51]. The presence of biofilms on prosthetic bases not only contributes to the development of denture stomatitis but also shortens the lifespan of these devices. Among these surface characteristics, studies have shown that contact angle is a predominant factor for bacterial adhesion, with a low contact angle (hydrophilic surface) being associated with reduced bacterial adhesion [51,52,53]. In contrast, surface roughness has not shown a significant correlation with bacterial adhesion [51]. Although all materials in this study exhibited significantly higher surface roughness in glazed samples compared to polished ones, the glazing surface treatment for MJT 3D-printed dentures can still be recommended for reducing bacterial adhesion and improving home care, as it results in a lower contact angle (more hydrophilic) than a polished surface.

The results for surface gloss are presented in Figure 8; all the glazed samples had significantly higher gloss units than the polished ones. These results are consistent with previous studies showing that glazing surfaces produce a higher gloss value than control groups [54,55]. Data on the surface gloss of milled versus 3D-printed denture base materials are limited. One study evaluated the surface gloss of a 3D-printed denture base material after polishing and coating treatments and found that the coating did not affect the surface roughness or gloss values [24]. These findings may be attributed to variations in the coating materials used. Based on these findings, it is recommended that clinicians consider glazing MJT 3D-printed denture materials to enhance surface gloss and improve surface wettability, which may facilitate patient hygiene and esthetic satisfaction. Additionally, because the color changes observed after glazing exceeded perceptibility and acceptability thresholds for some shades, it is advisable to use a glazed MJT shade guide during clinical shade selection to improve color-matching accuracy. These implications should be considered when integrating MJT-based workflows into clinical practice.

The limitations of this in vitro study include the inability to simulate all intraoral environments, such as using aging protocols, with different immersion liquids and periods [41,56]. While the same investigator (TN) performed the surface treatment procedures in a similar manner, human factors may still have introduced variations in the application technique. Moreover, the study did not fully address the potential inter-operator variability and the inherent subjectivity of manual glaze application, which may have affected surface outcomes. Measurement-related limitations also existed, particularly regarding gloss and wettability, which could be influenced by inconsistencies in sample positioning and the use of only a single type of spectrophotometry and glossmeter. Only two surface treatment protocols (polishing and polishing with glazing) and a single protective glaze coating were selected for this study, without a comparison with untreated controls or alternative finishing options, which restricted the generalizability of the findings. Furthermore, while the discussion suggested reduced bacterial adhesion due to increased surface hydrophilicity, no microbiological tests were conducted to support this implication. Future studies incorporating biofilm formation assays or in vivo analyses are necessary to validate these claims. Additional research is recommended to assess the optical and material properties of MJT-printed denture materials following simulated mechanical or thermal aging. Future investigations should also explore the influence of different glaze materials and application protocols, including variations in polymerization methods and durations. Comparative analyses between MJT-fabricated materials and those produced by other conventional, milling, or 3D-printing technologies are also warranted to aid clinicians in selecting the most appropriate materials and workflows for clinical use. Long-term clinical studies are essential to confirm and expand upon the present in vitro findings.

## 5. Conclusions

This in vitro study demonstrated that surface treatments significantly affected the color, gloss, surface roughness, and wettability of MJT 3D-printed denture materials. Glazing resulted in increased chroma, higher gloss values, and more hydrophilic surfaces compared to polishing alone. Although surface roughness increased after glazing, the esthetic and potential hygienic benefits support its application as a surface treatment for MJT-printed dentures. These findings underscore the importance of considering surface treatment protocols when evaluating the clinical performance of 3D-printed denture materials fabricated using MJT technology.

## Data Availability

The datasets presented in this article are not readily available because the data are part of an ongoing study and technical limitations. Requests to access the datasets should be directed to corresponding author.
